# ß-amylase1 mutant *Arabidopsis* plants show improved drought tolerance due to reduced starch breakdown in guard cells

**DOI:** 10.1093/jxb/erv323

**Published:** 2015-07-02

**Authors:** Christian Maximilian Prasch, Kirsten Verena Ott, Hubert Bauer, Peter Ache, Rainer Hedrich, Uwe Sonnewald

**Affiliations:** ^1^Biochemistry Division, Department of Biology, Friedrich-Alexander-University Erlangen-Nuernberg, Staudtstrasse 5, 91058 Erlangen, Germany; ^2^Institute for Molecular Plant Physiology and Biophysics, Biocenter, University of Wuerzburg, Julius-von-Sachs-Platz 2, 97082 Wuerzburg, Germany

**Keywords:** *Arabidopsis*, β-amylases, drought, guard cells, starch.

## Abstract

In plants, drought stress is a major growth limiting factor causing cell water loss through open stomata. In this study, guard cell-specific transcripts from drought-stressed *Arabidopsis* plants were analysed and a down-regulation of β-amylase 1 (BAM1) was found. In previous studies, BAM1 was shown to be involved in stomatal starch degradation under ambient conditions. Impaired starch breakdown of *bam1* mutant plants was accompanied by decreased stomatal opening. Here, it is shown that drought tolerance of *bam1* mutant plants is improved as compared with wild-type controls. Microarray analysis of stomata-specific transcripts from *bam1* mutant plants revealed a significant down-regulation of genes encoding aquaporins, auxin- and ethylene-responsive factors, and cell-wall modifying enzymes. This expression pattern suggests that reduced water uptake and limited cell wall extension are associated with the closed state of stomata of *bam1* mutant plants. Together these data suggest that regulation of stomata-specific starch turnover is important for adapting stomata opening to environmental needs and its breeding manipulation may result in drought tolerant crop plants.

## Highlight

*bam1* mutant plants impaired in stomatal starch degradation showed an improved drought tolerance associated with a down-regulation of guard cell-specific gene expression involved in water uptake and cell expansion.

## Introduction

Water availability is the most limiting factor for plant growth. To avoid yield losses, irrigation is common practice in agriculture in dry areas worldwide. This leads to a massive water consummation of agriculture—globally, agriculture is responsible for more than 70% of freshwater withdrawal (FAO, 2014). This level can hardly be maintained and cannot be increased in the future. Considering expected climate changes as well as an increased food demand to feed a growing world population, this sets a new challenge for breeding. Crop plants performing well under sub-optimal environmental conditions and on marginal lands are urgently needed. Thus improving irrigation systems and breeding for crop plants with better drought tolerance will be essential to secure food production in the future. Understanding of regulatory networks coordinating stomatal transpiration and soil water use efficiency (WUE) will be one strategy. Soil water is taken in by the root system and lost from the plant body via stomata, when open, to allow CO_2_ intake. During photosynthesis atmospheric CO_2_ is converted to organic carbon molecules mainly in mesophyll cells. To optimize water loss and CO_2_ gain, cross talk exists between guard cell ion transport and metabolism on the one hand and mesophyll photosynthesis on the other hand (Raschke and Hedrich, 1985; Hedrich, 2012; Lawson and Blatt, 2014). Stomata conductance is regulated by turgor changes of guard cells. Increased turgor pressure results in stomatal opening, while turgor reduction results in stomatal closure. One major regulator of stomatal closure is the water stress hormone abscisic acid (ABA; Christmann *et al.*, 2006). Under drought stress conditions, a tripartite complex composed of ABA, its receptor PYL/PYR/RCAR and ABA Insensitive (ABI1) is formed, which inhibits the negative regulators of ABA signalling (Raghavendra *et al.*, 2010). This allows phosphorylation and activation of downstream targets. One target is sucrose non-fermenting 1-related subfamily 2 protein kinase (SnRK2), which is able to regulate S-type anion channels such as SLAC1 (slow anion channel-associated 1; Geiger *et al.*, 2009; Roelfsema *et al.*, 2012 for review). SLAC1 activation results in an anion efflux and a depolarization of the plasma membrane activating the gated outward rectifying K^+^ channel GORK, which leads to an enhanced potassium efflux and stomatal closure (Hedrich, 2012). While the role of ion transport in stomatal movements is widely accepted, the potential role and regulation of guard cell sugar metabolism in this process is still under debate (Lawson *et al.*, 2009). Besides the uptake and release of potassium salts, the interconversion of starch and malate builds the osmotic motor that drives stomata movement (Outlaw and Manchester, 1979; Schnabl, 1980; Hedrich, 2012). Malate is gluconeogenically converted into starch, which also supports stomatal closure. One of the first steps during guard cell opening is the conversion of starch to the disaccharide maltose (reviewed in Daszkowska-Golec and Szarejko, 2013). In plants this reaction is catalysed by β-amylases (BAMs; Weise *et al.*, 2005). In *Arabidopsis* nine β-amylase-like proteins are known; four of them are targeted to chloroplasts (Lao *et al.*, 1999; Sparla *et al.*, 2006; Fulton *et al.*, 2008). Several studies in different plant species indicated that BAMs exhibit a complex regulation, in which the expression and activity of BAMs is mainly influenced by sugars (Mita *et al.*, 1995; Maeo *et al.*, 2001), phytohormones (Wang *et al.*, 1996), and different abiotic stress factors—particularly light (Datta *et al.*, 1999; Tepperman *et al.*, 2001), cold (Kaplan and Guy, 2004; Kaplan and Guy, 2005; Maruyama *et al.*, 2009; Sicher, 2011), salt (Dreier *et al.*, 1995; Datta *et al.*, 1999), osmotic (Dreier *et al.*, 1995; Valerio *et al.*, 2011), and heat stress (Dreier *et al.*, 1995; Kaplan and Guy, 2004; Monroe *et al.*, 2014). Interestingly, *bam1* knockout mutant plants show increased amounts of starch in illuminated guard cells, resulting in reduced stomata opening (Valerio *et al.*, 2011). Apart from maltose, glucose represents another important product of starch breakdown, regulating stomatal movements. Antunes *et al.* (2012) demonstrated guard cell-specific sucrose hydrolysis to be involved in regulation of stomatal conductance. By guard cell-specific down-regulation of sucrose synthase 3 in transgenic potato plants the authors could show a reduced stomatal conductance while WUE increased with decreasing sucrolytic activity. Increasing the sucrose hydrolytic activity by stomata-specific expression of yeast invertase led to the opposite effect. This study clearly demonstrates an important role of sucrose utilization in regulation of stomatal conductance. How this regulation is exerted remains elusive. Beside sugar metabolism, also sugar sensing seems to be involved. This hypothesis has been supported by the observation that overexpression of hexokinase in guard cells promotes stomatal closure (Kelly *et al.*, 2013). Although it is not intuitive why reduced sucrose utilization and increased hexokinase activity would both lead to stomatal closure, the results clearly demonstrate the importance of sugar metabolism/sensing in regulation of stomata conductance.

To investigate molecular and metabolic processes involved in the coordination of stomata conductance under drought stress, guard cell-specific transcript networks were determined by microarray analysis and compared with whole leaf transcript profiles. The results indicate a shift in sucrose utilization in favour of guard cells and increased expression of genes involved in starch synthesis, while starch degradation appeared to be down-regulated. In particular, BAM1 was strongly down-regulated. To functionally validate the role of BAM1 down-regulation during drought, growth performance of *bam1* mutant plants under drought stress was monitored.

*Bam1* mutant plants showed a significant increase in drought tolerance associated with a strong down-regulation of genes involved in water uptake and cell expansion. These results indicate that starch turnover is important in regulating stomata aperture and that inhibiting BAM1-mediated starch breakdown in guard cells can lead to improved plant performance under drought stress.

## Materials and methods

### Plants and growth conditions

In this work *Arabidopsis thaliana* (*Arabidopsis*) wild-type plants (ecotype Columbia) were used as well as a T-DNA insertion line *bam1*, a homozygous line without any gene expression of BAM 1 (shown by Fulton *et al.*, 2008); for these experiments data were confirmed, see Supplementary Figure S1 at *JXB* online). Microarray analysis revealed a raw value reduction from 3441 in leaves of Col-0 plants to 148 in leaves of *bam1* mutant plants. The same reduction can be found in guard cells, given by a reduction from 9303 in guard cells of Col-0 to 297 in guard cells of *bam1* plants (see Supplementary Table S1 at *JXB* online). After 2 d overnight 4 °C treatment for stratification, plants were grown on soil under short-day conditions (8h light, 16h dark), 60% humidity, and 22 °C (day) or 18 °C (night). Fourteen days later young seedlings were further cultivated in single plant pots containing 160g soil and plants were watered with defined volumes of water as described by Prasch and Sonnewald (2013). Controlled plant cultivation was achieved by a temperature regime following the light/dark cycle with 22 °C/18 °C and a diurnal rhythm of 12h of light at approximately 80 µmol m^−2^ s^−2^ and exactly 60% humidity at day and night provided by a plant climate chamber (Plant-Master PGR 3045, CLF Plant Climatics GmbH, Germany). Each stress treatment was performed in independent plant cultivations. The drought stress experiment was repeated four times, showing an increase in fresh weight of at least 14%. Therefore 5–6 week old plants were exposed to mild drought stress for 5 d. Harvest of plant material was realized at the end of the light period. After determining the fresh weight of each plant, stomata-specific RNA was prepared.

### Application of mild drought

Mild drought stress was applied according to Prasch and Sonnewald (2013). In brief, a Decagon Devices sensor allowed controlling water amounts of each plant pot containing 160g soil. The correlation curve, showing weight over field capacity, was used to define the area for control and drought stress conditions. The upper limit was given at a soil moisture content of 100%, whereas the lowest value was given by drying the soil overnight. Values in between were determined by adding 10ml each. Control plants were watered daily according to 55% of the correlation curve with a deviation of 10%. Mild drought stress was given by 30% with a deviation of 10%, achieved by withholding water. If necessary, plants were watered with low amounts of water. Reduced water potential of *Arabidopsis* plants under these conditions has already been shown (Prasch and Sonnewald, 2013). Humidity was kept at 60% and water supply was guaranteed for control plants.

### Sampling, stomata-specific RNA extraction

After plants had been treated with different stresses, the fresh weight was measured of each plant 6–10h after the beginning of the light period. In parallel, 20 plants of each condition and each genotype were dried at 80 °C for 3 d for determination of plant dry weight. The stomata extraction procedure was performed as described by Bauer *et al.* (2013). In brief, *Arabidopsis* leaves of Col-0 plants and *bam1* mutant plants were harvested and major veins were removed. Together with ice-cold deionized water and crushed ice, the remaining leaf material of five to six plants for each replicate was placed into a blender. Epidermal peels were isolated within 8min by successive blender cycles of 1–2min each. After two rounds of blending, the suspension was added to a 210 µm nylon mesh. Epidermal factions were collected from the mesh and further processed for RNA isolation. The reliability of the method was tested by neutral red staining of viable cells and qPCR analysis of guard cell and vascular tissue marker mRNAs (Bauer *et al.*, 2013). After sampling, material was immediately frozen in liquid nitrogen. In addition, leaf material was stored at −80 °C for further analysis. Stomatal RNA was isolated using the RNeasy Plant Mini Kit (www.quiagen.com) according to the manufacturer’s protocol. Two to three biological replicates were used for microarray hybridization.

### PCR analysis

Total RNA was extracted as described by Logemann *et al.* (1987) for cDNA synthesis. From 1.25 μg of total RNA, cDNA was synthesized according to Biemelt *et al.* (2004). The following oligonucleotides were used for RT-PCR: BAM1 forward primer, 5′-AGAACGTATAGAGAAGGAGGGATTG-3′; BAM1 reverse primer, 5′-CCGTCTCTGAACCTTGTGTTGTAGTA-3′; Ubiquitin was used as internal control—UBQ forward primer 5′-ATGCAGATYTTTGTGAAGAC-3′; UBQ reverse primer 5′-ACCACCACGRAGACGGAG-3′. Genomic DNA was extracted as described (Edwards *et al.*, 1991). The following oligonucleotides were used for PCR on genomic DNA: SALK_039895_BAM1_RP, 5′-CGCTTAATTTATCGCATCAGC-3′; T-DNA-Primer_LB, 5′-ATTTTGCCGATTTCGGAAC-3′.

### Microarray hybridization

For transcript profiling, RNA was purified using RNeasy Mini Spin Columns (Qiagen) following the manufacturer’s protocol. Quality and quantity was measured with an Agilent RNA 6000 Nano Chip on an Agilent 2100 BioAnalyzer (version B.02.03 BSI307) following by Agilent RNA 6000 Nano Assay Protocol2. Sample labelling and preparation for microarray hybridization were performed as described in the one-colour microarray-based gene expression analysis protocol—provided by Agilent—including the one-colour RNA spike-in kit (Agilent Technologies). Cy3-labelled samples were fragmented and loaded on the array (Agilent Arabidopsis V4 – Design number 21169), hybridized overnight (17h/65 °C), and washed. Slides were scanned on the Agilent Microarray Scanner with extended dynamic range at high resolution.

### Data extraction and analysis

The feature extraction software (Version 11.7.1; Agilent Technologies) generated text files for further data analysis. Those text files were imported into GeneSpring GX 12.6.1 (Silicon Genetics). For each experiment replicates of stressed plants were compared with their respective controls. Microarray data were log_2_-transformed followed by normalization to Quantil and corrected to the median of its value across the data set. After applying a One-way ANOVA (*P*≤0.05, variance assumed as equal) test, Volcano plot analysis identified statistically significant (*P*≤0.05; equal variances assumed), more than two-fold differentially expressed genes between two conditions. Two to three replicates were analysed for each genotype. Functional assignment was performed based on annotations given by MAPMAN bins (http://mapman.gabipd.org/web/guest/mapman). Therefore the percentage of significantly regulated *features* from a specific functional group was presented compared with the percentage of *features* from that specific group to the entire chip. To make stringent the structure of categories, the category ‘gluconeogenesis’ was assigned to ‘minor carbohydrates’, and the category ‘biodegradation of Xenobiotics’ to the category ‘not assigned’. Arrays have been deposited under the accession number GSE59321 in the National Center for Biotechnology Information Gene Expression Omnibus database.

### Analysis of starch content

Starch content of leaves was determined according to Stitt *et al.* (1989). To analyse the size of starch granules in stomata of Col-0 and *bam1* plants, starch content was visualized by iodine staining. Two hours after light induction, leaf samples of 6-week old plants were decolorized with hot 80% (v/v) ethanol at 80 °C for 20min and stained with LUGOL solution (2% iodine and 10% KI). Subcellular localization of starch grains was observed using a Leica DMR- microscope (Bensheim) and the corresponding software SPOT ADVANCED^TM^. More than 80 stomata of wild-type and the *bam1* mutant were randomly selected from different plants. According to Valerio *et al.* (2011) the amount of starch per guard cell was calculated as the total pixel area of the dark brown circles isolated in each cell using Photoshop CS5.

### Stomatal aperture measurement

For stomatal measurements, wild-type and the *bam1* mutant plants were grown in parallel under ambient and stress conditions. Two hours after light induction the stomata on the abaxial side of *Arabidopsis* leaves were fixed using nail polish and imprints were immediately analysed using a Leica DMR light microscope (Bensheim). The software SPOT ADVANCED^TM^ allowed the calculation of both length and width of randomly selected stomata on the digital images. Stomatal apertures were calculated as the ratio between the width and length of the pore (more than 80 stomata were analysed for each genotype and each condition).

## Results and discussion

### Identification of stomata-specific transcripts of drought-stressed *Arabidopsis* plants

**Fig. 1.**
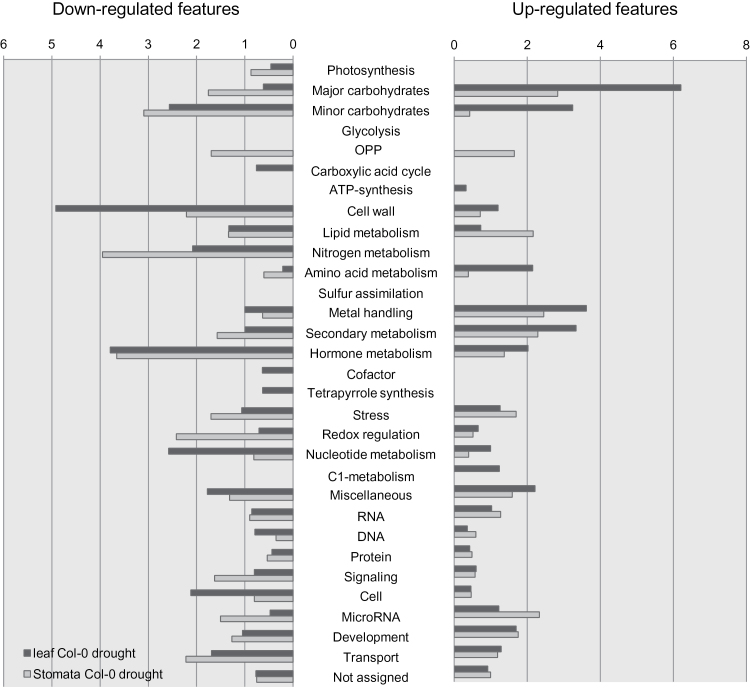
Functional assignment of up- and down-regulated stomata-specific *features* regulated by water stress in Col-0 plants. After 27-d-growth under control conditions, *Arabidopsis* Col-0 plants were exposed to mild drought stress for 5 d (see materials and methods). Subsequent microarray analysis identified 1668 *features* to be differentially regulated under drought stress in leaves and 1076 *features* to be differentially regulated under drought stress in stomata in comparison with plants under control conditions. Those were divided into up- (674 genes in leaf and 542 in stomata) and down- (994 genes in leaf and 534 in stomata) regulated groups and grouped according to a classification of *features* based on bins provided by MAPMAN. Bars illustrate the percentage of significantly regulated *features* from a specific functional group relative to the percentage of *features* from that specific group to the entire chip. Black bars represent transcripts of drought-stressed leaves in Col-0; grey bars show stomata-specific transcripts of drought-stressed Col-0 plants. (A colour version of this figure is available at *JXB* online.)

Despite the large number of studies investigating short-term responses of guard cells to water loss, relatively little is known about adaptation mechanisms to long-term drought stress. To gain deeper insight into the molecular responses controlling stomatal aperture, stomata-specific and whole leaf transcription profiles of *Arabidopsis thaliana* Col-0 plants, kept for 5 d under moderate drought stress conditions (as described in Prasch and Sonnewald, 2013), were analysed. Guard cell preparation and RNA isolation was done as described in Bauer *et al.* (2013). RNA from guard cells and whole leaves was subsequently hybridized to whole-genome Agilent microarrays and analysed for significantly, differentially expressed genes. After conducting an ANOVA-Test (22995 significant *feature*s), comparative analysis identified 1076 more than two-fold differentially expressed *features* enriched in guard cells and 1668 differentially expressed *features* in leaves of drought-stressed plants compared with control plants kept under ambient and well watered conditions. Gene lists can be found in Supplementary Table S1 (available at *JXB* online). Differentially expressed transcripts were subdivided into up- and down-regulated ones and classified according to MAPMAN categories (mapman.gabipd.org/web/guest/mapmanstore; Thimm *et al.*, 2004; Fig. 1; for detail see Supplementary Table S2 at *JXB* online). Among the up-regulated mRNA pool, transcripts involved in carbon metabolism were most affected both in leaf and stomata-specific fractions. Furthermore, the categories metal handling, secondary metabolism, hormone metabolism, and miscellaneous were enriched in drought-stressed leaves and to a lesser extent in stomata, too. Minor carbohydrates and amino acids were only represented in leaves. By contrast, *features* such as microRNA, stress, lipid metabolism, and oxidative pentose phosphate pathway (OPP) were up-regulated in stomata-specific samples. Among the down-regulated transcripts those associated with cell wall, nucleotide metabolism, and cell were strongly enriched in whole leaf samples, whereas major carbohydrates, OPP, N-metabolism, secondary metabolism, redox-regulation, and signalling were mainly down-regulated in stomata-specific samples. Furthermore, genes encoding transport proteins were down-regulated in both samples. In this context, ion channels were not significantly altered under long-term drought stress conditions (see Supplementary Figure S2 at *JXB* online).

Given that ABA is key in activating drought resistance responses (Finkelstein and Gibson, 2002; Yamaguchi-Shinozaki and Shinozaki, 2006; Nakashima *et al.*, 2009), the functional category ‘hormone’ was analysed in more detail. Leaves of drought-stressed Col-0 plants showed a marked increase of the subcategory ABA as >40% of the differentially up-regulated *features* corresponded to ABA associated genes (Supplementary Table S3). For guard cells, it has recently been reported that ABA synthesis can occur in a cell-autonomous manner to induce stomata closure (Bauer *et al.*, 2013). Under drought stress, 25% of the up-regulated hormone genes could be linked to ABA indicating increased levels of ABA in guard cells. Glucosidase 1 (BG1), an enzyme that cleaves stored ABA glucosides and provides active ABA upon stress (Lee *et al.*, 2006), showed increased transcript levels in drought-induced guard cells (Supplementary Table S3). These transcriptional changes suggest that under this condition ABA might be released form the vacuolar store and potentially activates SNF1-related protein kinase 2 (SnRK2). In support of this hypothesis, most of the published SnRK2 target genes (Mizoguchi *et al.*, 2010) in guard cells were found to be up-regulated under water limiting conditions (Supplementary Table S4).

### Genes involved in guard cell starch metabolism respond to drought stress

**Fig. 2.**
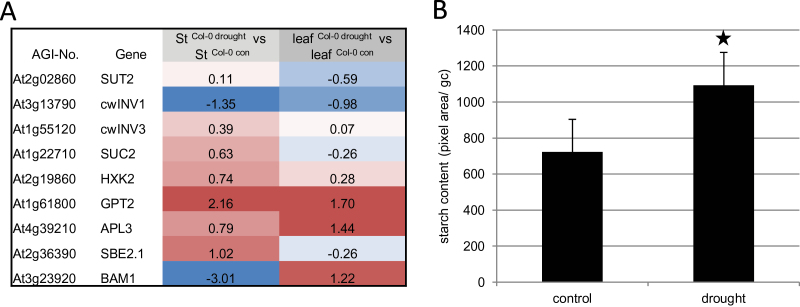
Selection of carbohydrate-associated genes significantly regulated, and starch content of guard cells under control and drought stress conditions. (A) Transcriptional changes of genes associated with carbohydrate fluxes determined by microarray analysis either in leaves or in guard cells of drought-stressed *Arabidopsis* plants. Drought-induced expression levels were compared with the expression levels under control conditions. Log_2_ values are given as fold change compared with control plants. The colours saturate at 1.3-fold change. St, stomata. Red represents an increase and blue represents a decrease in transcript levels. See also Supplementary Table S4 available at *JXB* online. (B) Via digital image processing starch content was quantified as the total pixel area of starch accumulations visible in chloroplasts of single guard cells in Col-0 plants under control and drought stress conditions. Values are means of more than 75 guard cells. Statistically significant differences were determined using a two-tailed *t* test assuming a normal distribution and are indicated by asterisks (*P*, 0.05). (A colour version of this figure is available at *JXB* online.)

The significant enrichment of the category ‘major carbohydrates’ points to the involvement of sugars and starch in the regulation of stomatal aperture (Antunes *et al.*, 2012). Therefore, processes associated with assimilate allocation were analysed in more detail. A reduced expression of sucrose transporter 2 (SUT2) and cell-wall bound invertase 1 (cw-Inv1; see Fig. 2A and all data in Supplementary Table S5 available at *JXB* online) in leaf samples, but up-regulation of sucrose-proton symporter 2 (SUC2), hexokinase 2 (HXK2), and cw-Inv3 in the guard cell fraction was observed. Moreover, enhanced expression of glucose-6-phosphate/phosphate translocator 2 (GPT2) could be found. Since glucose-6-phosphate is the preferred substrate for starch synthesis in guard cells (Overlach *et al.*, 1993) this suggests a redirection of metabolites in favour of starch synthesis. This assumption is furthermore supported by increased transcript levels of ADP-glucose pyrophosphorylase 3 (APL3) and starch branching enzyme 2 (SBE2) in guard cells. These transcriptional data are paralleled by the observed increase in stomatal starch in drought-stressed Col-0 plants as compared with the control condition (see Fig. 2B). Altogether, these observations suggest an improved conversion of soluble sugars to starch, which would lead to a reduced osmotic potential, water efflux and hence stomata closure under drought stress conditions.

Although some genes involved in starch degradation were marginally up-regulated (Supplementary Table S5), out of the nine genes encoding β-amylases and β-amylase-like proteins a strong down-regulation for BAM1 (eight-fold) and BAM5 (seven-fold) could be observed, suggesting inhibition of amylolytic starch degradation under drought stress in guard cells. To the best of the authors’ knowledge no data exist describing a potential role of BAM1 in drought stress. Based on microarray experiments, BAM1 is the predominate β-amylase expressed in guard cells (Supplementary Table S6). Furthermore, BAM1 is known to be involved in the chloroplastic starch degradation and release of maltose (Sparla *et al.*, 2006). Valerio *et al.* (2011) have shown that *Arabidopsis* mutants lacking BAM1 contained more starch in guard cells and stomata were more tightly closed compared with wild-type plants. From this scenario one may assume that starch degradation in guard cells of plants exposed to drought stress is reduced.

### *bam1* mutant showed improved drought tolerance

**Fig. 3.**
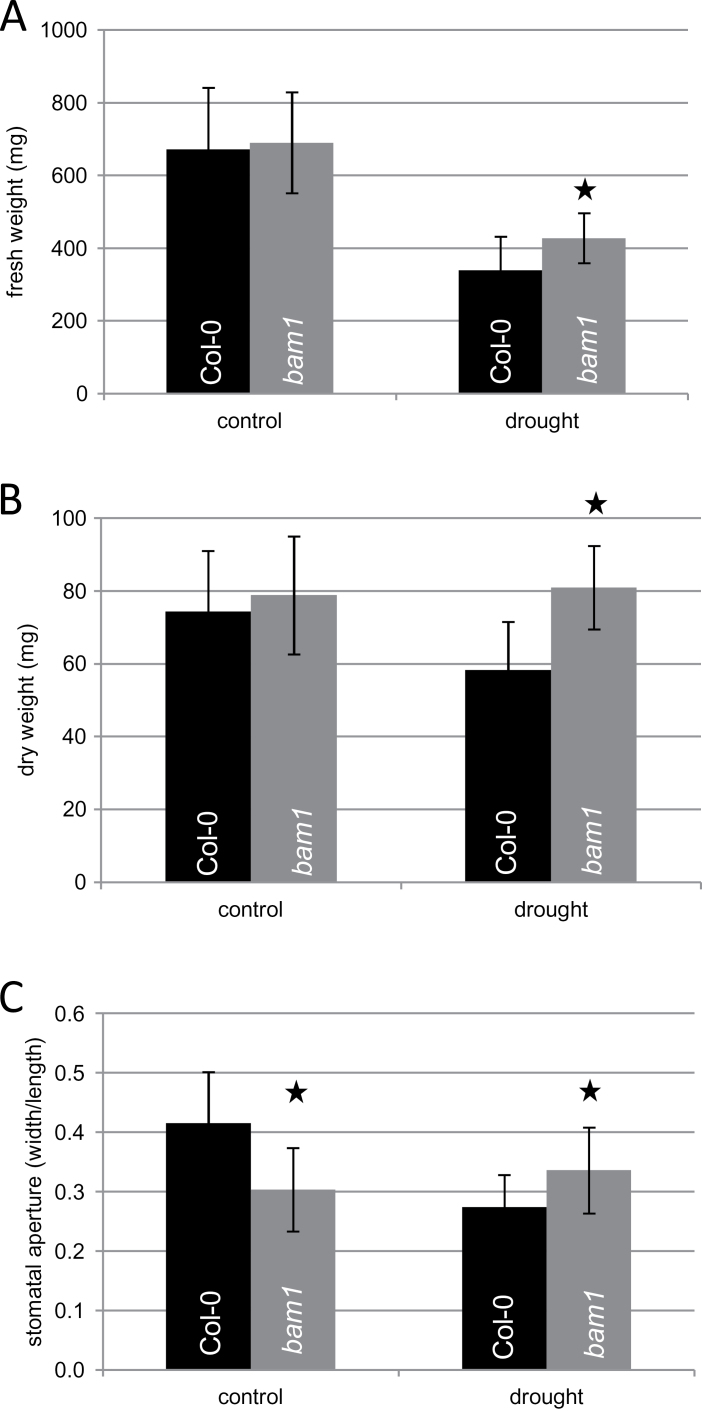
Biomass determination and stomatal aperture analysis of wild-type and *bam1* mutant plants under drought compared with control conditions. (A) Fresh weight of whole rosettes from drought-stressed (5 d of stress treatment) plants and 34-d-old control plants for each genotype. Data points represent an average of 20 plants ± SD. Statistically significant differences from Col-0 plants were determined using a two-tailed *t* test assuming a normal distribution. Statistically significant differences are indicated by asterisks (*P*, 0.05). (B) Dry weight of whole rosettes from drought-stressed (5 d of stress treatment) and 34-d-old control plants for each genotype. Data points represent an average of 20 plants ± SD. Statistically significant differences from Col-0 plants were determined using a two-tailed *t* test assuming a normal distribution. Statistically significant differences are indicated by asterisks (*P*, 0.05). (C) Stomatal aperture measurement was calculated as the ratio between width and length of the stomatal pore. Values are given as means of more than 80 stomata for each sample. (A colour version of this figure is available at *JXB* online.)

**Fig. 4.**
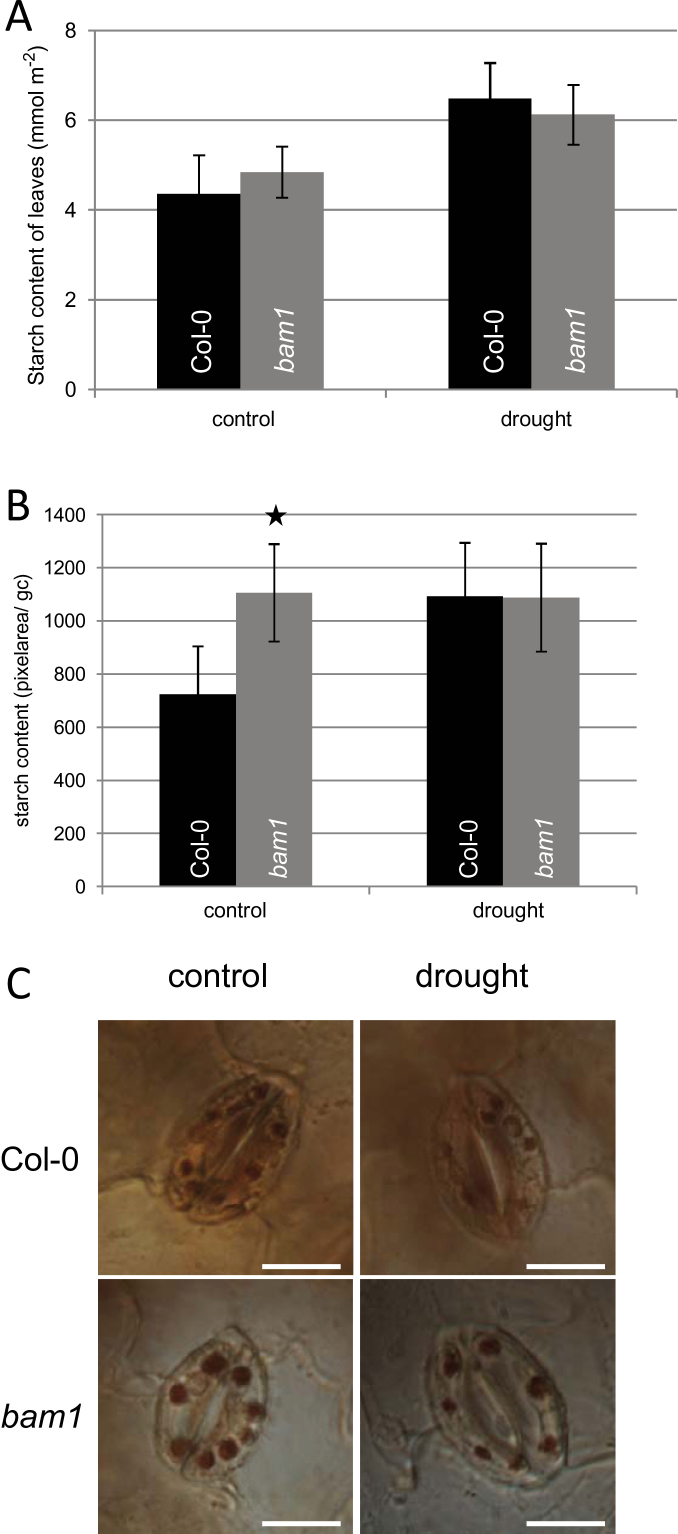
Starch content of whole leaves and guard cells from Col-0 and *bam1* mutant plants under control and drought conditions. (A) Starch content of leaves under control and drought stress conditions were measured in the indicated *Arabidopsis* genotypes. (B) Via digital image processing starch content was quantified as the total pixel area of starch accumulations visible in chloroplasts of single guard cells in Col-0 and *bam1* plants under different control and drought stress conditions. Values are means of more than 75 guard cells. Statistically significant differences from Col-0 plants were determined using a two-tailed *t* test assuming a normal distribution. Statistically significant differences are indicated by asterisks (*P*, 0.05). (C) Iodine staining allowed visualization of starch accumulation in wild-type and *bam1* guard cells under control conditions. Bars represent 10 µm. (A colour version of this figure is available at *JXB* online.)

Assuming BAM1 to be the major β-amylase responsible for starch degradation in stomata, drought-induced inhibition of BAM1 would inhibit starch turnover and synthesis of soluble carbohydrates. Therefore, the question of whether loss of BAM1 function would affect stomatal aperture under drought stress was addressed. Col-0 and *bam1* mutant plants (Valerio *et al.*, 2011; Supplementary Figure S1; microarray data, Supplementary Table S1) were grown under control conditions for 29 d followed by 5 d of drought stress according to Prasch and Sonnewald (2013). Under control conditions the biomasses of Col-0 plants and *bam1* plants were indistinguishable (see Fig. 3A). However, drought-stressed b*am1* plants produced 25% more fresh weight, and even 38% more dry weight as compared with Col-0 plants (Fig. 3B). To score the stomata opening, the stomatal aperture, given by the width to length ratio, of both Col-0 and *bam1* plants, was measured. When subjected to drought, stomata closure can be seen for Col-0 plants (Fig. 3C). Consistent with earlier observations by Valerio *et al.* (2011), *bam1* mutants showed reduced stomata opening under control conditions as compared with Col-0 plants (Fig. 3C). Under drought stress conditions, however, stomata of *bam1* mutant plants did not respond and therefore were more opened than in Col-0 plants. The overall starch content of control or drought-stressed leaves was unaltered in *bam1* mutant plants (Fig. 4A) as compared with Col-0. However, guard cells of *bam1* knockout plants exhibited larger starch granules than Col-0 under control conditions (Fig. 4B, C). Under drought conditions, starch content remained high in stomata of *bam1* mutant plants. Thus inhibiting starch turnover in *bam1* mutant plants improves drought tolerance and biomass production.

### Down-regulation of cell wall-modifying enzymes, aquaporins, and auxin response factors in guard cells of *bam1* mutant plants under drought stress

**Fig. 5.**
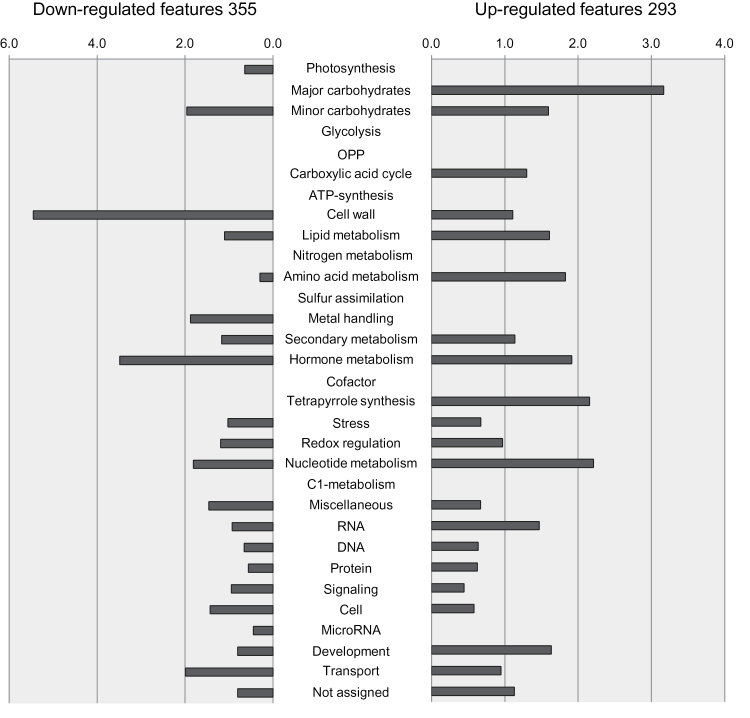
Functional assignment of up- and down-regulated stomata-specific *features* of *bam1* plants exposed to drought stress. Microarray analysis of stomata-specific transcripts identified 648 differentially regulated genes in *bam1* plants, which cannot be found in stomata of Col-0. Up- and down-regulated transcripts were separated and grouped according to a classification of *features* based on bins provided by MAPMAN. Bars illustrate the percentage of significantly regulated *features* from a specific functional group relative to the percentage of *features* from that specific group to the entire chip. (A colour version of this figure is available at *JXB* online.)

Stimulated by the improved drought tolerance of *bam1* mutant plants stomata-specific processes were studied in more detail by transcript profiling. A comparison of stomata-specific transcripts of Col-0 under drought stress (1076 *features*) and stomata transcripts of *bam1* plants (1082 transcripts) identified 648 genes, specifically at least two-fold regulated, in guard cells of *bam1* plants under drought stress conditions (Supplementary Tables S1 and S7). Within the category of up-regulated genes, a classification according to MAPMAN showed enrichment in carbohydrates, synthesis of tetrapyrroles, and nucleotide metabolism (see Fig. 5; for detailed information about the categories see Supplementary Table S8 available at *JXB* online). Noticeable, among the transcripts of *bam1* mutants ‘not assigned’ more than 30 pentatricopeptide repeat (PPR) family proteins appeared (Supplementary Table S7). The *Arabidopsis* genome contains more than 450 PPRs, but most of them are still uncharacterized (Schmitz-Linneweber and Small, 2008; Laluk *et al.*, 2011).

Among the down-regulated genes, members of the category ‘cell wall’ were found to be overrepresented (Supplementary Table S7). In this category genes encoding arabinogalactan proteins, expansins, and Xyloglucan:xyloglucosyl transferases were enriched and approximately three-fold down-regulated. Arabinan has an essential function in guard cell movement (Jones *et al.*, 2003; Seifert and Roberts, 2007), and xyloglucans have been reported to influence the strength and elongation of cell walls (Goujon *et al.*, 2003; Vissenberg *et al.*, 2005). This implies that guard cells from *bam1* mutants are characterized by a more rigid cell wall. This hypothesis is supported by the down-regulation of several auxin-dependent transcripts (Supplementary Table S7). Assuming that the cell wall modifications would limit water uptake, a reduced expression of aquaporins would be expected. As shown in Supplementary Table S7, several aquaporins are indeed more than two-fold down-regulated in guard cells of water stressed *bam1* plants.

In summary, data on stomata-specific drought-stressed *bam1* plants identified genes related to starch–sugar balance, cell wall modification, and water transport. These alterations are likely to affect guard cell osmotic/turgor pressure, water movement, and cell wall extension driving the guard cell hydraulics towards stomatal closure.

## Supplementary data

Supplementary data are available at *JXB* online.

Figure S1. Molecular analysis of the *bam1 Arabidopsis* T-DNA insertion mutant.

Figure S2. Expression of ion- and water channels in stomata of Col-0 plants under drought stress conditions.

Table S1. Gene expression of leaves and guard cells of drought-stressed Col-0 and *bam1* plants.

Table S2. Functional categorization of transcripts of drought-stressed Col-0 plants.

Table S3. Gene expression of hormone-associated transcripts in drought-stressed Col-0 plants.

Table S4. Gene expression of leaves and guard cells of SNRK2-regulated genes in drought-stressed Col-0 plants.

Table S5. Gene expression of transcripts associated with central metabolism/sugar/starch metabolism of leaves and guard cells in drought-stressed Col-0 and *bam1* plants.

Table S6. Raw data and differentially expressed genes of β-amylases in guard cells under drought in Col-0 and *bam1* plants.

Table S7. 648 *bam1* specific guard cell transcripts of drought-stressed *bam1* plants.

Table S8. Functional categorization of transcripts of drought-stressed *bam1* mutant plants.

Supplementary Data

## Abbreviations:

ABA: abscisic acid
ABI1: ABA Insensitive 1
APL3: ADP-glucose pyrophosphorylase3
BAM: β-amylase
BG1: β-glucosidase1
cw-Inv: cell-wall bound invertase
GORK: gated outwardly-rectifying K+ channel
GPT2: glucose-6-phosphate/phosphate translocator2
HXK2: hexokinase2
OPP: oxidative pentose phosphate pathway
SBE2: starch branching enzyme2
SLAC1: slow anion channel-associated 1
SnRK2: sucrose non-fermenting 1-related subfamily 2 protein kinase
SUC2: sucrose-proton symporter2
SUT2: sucrose transporter2
WUE: water use efficiency.
