# Supplementary material for: ß-amylase1 mutant Arabidopsis plants show improved drought tolerance due to reduced starch breakdown in guard cells
Source: J Exp Bot. 2015 Jul 2;66(19):6059–67. doi: 10.1093/jxb/erv323 (PMC4566991; doi:10.1093/jxb/erv323)
Supplement: Supplementary Data [file supp_66_19_6059__index.html]

ß-amylase1 mutant Arabidopsis plants show improved drought tolerance due to reduced starch breakdown in guard cells — ß-amylase1 mutant Arabidopsis plants show improved drought tolerance due to reduced starch breakdown in guard cells — Supplementary Data 

# ß-amylase1 mutant *Arabidopsis* plants show improved drought tolerance due to reduced starch breakdown in guard cells

## Supplementary Data

Data files

- Supplementary Data - Supplementary Data
- Supplementary Data - Supplementary Data
- Supplementary Data - Supplementary Data
- Supplementary Data - Supplementary Data
- Supplementary Data - Supplementary Data
- Supplementary Data - Supplementary Data
- Supplementary Data - Supplementary Data
- Supplementary Data - Supplementary Data
- Supplementary Data - Supplementary Data
