# Supplementary material for: ß-amylase1 mutant Arabidopsis plants show improved drought tolerance due to reduced starch breakdown in guard cells
Source: J Exp Bot. 2015 Jul 2;66(19):6059–67. doi: 10.1093/jxb/erv323 (PMC4566991; doi:10.1093/jxb/erv323)
Supplement: Supplementary Data [file supp_erv323_jexbot148692_file009.pdf]

ß--amylase1 mutant Arabidopsis plants show improved drought tolerance due to reduced starch breakdown in guard cells

Christian Maximilian Prasch, Kirsten Verena Ott, Hubert Bauer, Peter Ache, Rainer Hedrich, and Uwe Sonnewald

Supplemental Files

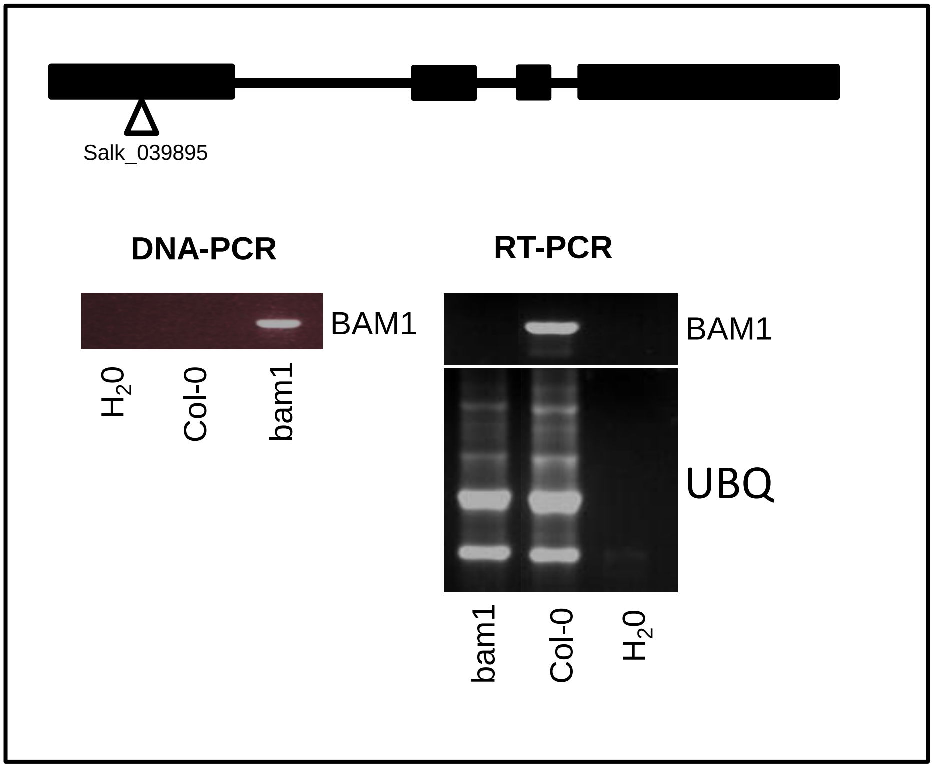

Supplementary figure S1: Molecular analysis of the *bam1* Arabidopsis T-DNA insertion mutant. PCR on genomic DNA revealed an insertion in the BAM1 locus not detectable in Col-0 plants (Primer: LB1.3 ATTTTGCCGATTTCGGAAC, RP, CGCTTAATTTATCGCATCAGC) Expression of BAM1 transcript was shown by RT-PCR with gene-specific primer sets. Ubiquitin (UBQ) served as an internal control.

Gene	d (Col-0)
AHA1	-0.38
AHA2	-0.4
AHA3	0.17
AHA4	0.66
AHA5	-0.55
AHA6	n.d.
AHA7	n.d.
AHA8	-0.01
AHA9	-1.08
AHA10	n.d.
AHA11	-0.36
AHA12	n.d.
KAT1	-0.7
KAT2	-0.84
AKT1	0.47

	Gene	d (Col-0)
Ca-channel	ATTPC1	n.d.
S-type anion channel	ATCLC-A	-0.42
	CLC-B	-0.43
	CLC-E	-0.48
	CLC-D	0.42
	AT5G33280	n.d.
	CLC-C	-0.2
	CLC-F	-0.1
K-channel	GORK	-0.03

Supplementary figure S2: Expression of Ion- and water channels in Stomata of Col-0 plants under drought stress conditions. Microarray-analysis of stomata-specific RNA of drought-stressed Col-0 plants have been performed. Transcriptional changes of ion- and water channels associated with stomata opening and closure are visualized. Genes corresponding to stomata processes are according to Daszkowska-Golec and Szarejko, 2013. Log_2values of the fold change compared to control plants. The colors saturate at 1.3-fold change. Red represents an increase and blue represents a decrease in transcript levels. Drought (d), control (con), n.d., not determined.
